# Editorial Notes

**Published:** 1938

**Authors:** 


### Editorial Notes

**Development of Thoracic Surgery.**

The development of thoracic surgery has been slow in comparison with that of other regions. Perhaps the reason (? explanation) is that the physics of lung expansion have been misconceived and the importance of atmospheric and intrapleural pressure overrated. Yet it is more than forty years since Sir William Macewen removed a whole lung for phthisis. The modest but dramatic way in which he announced this surgical achievement at the International Congress of Medicine in 1913 is unforgettable. The seventeenth Congress was held in London and on August 12th, 1913, Professor F. Sauerbruch opened a discussion on intrathoracic surgery.[1] In the course of his remarks Professor Sauerbruch explained the various methods which had been adopted to maintain the difference of pressure between the air inside and outside the lungs when the thorax had been opened. He described the somewhat elaborate pressure chamber which he had devised in order to render a free open thoracotomy safe.

Professor Tuffier sent a communication which was read in his absence. He had come to the conclusion, he wrote, that the danger of an operative pneumothorax had been overrated, at least if the pneumothorax had been slowly produced.

Then followed Professor William Macewen, of Glasgow. He said he wished to draw attention to two points. The first was the result he had obtained in removal of one lung for phthisis, and of this operation he had had four cases. One of these was shown (at the Congress) in excellent health eighteen years after the operation. The

[1] From the *Lancet*, 1913, ii. p. 551

second point related to the physics of the chest. He said it was usually taught that the lung remained in contact with the chest wall in consequence of the atmospheric pressure, but he did not accept this explanation. He showed by experiment that if two pieces of glass or rubber are pressed into contact they cannot be separated even when the atmospheric pressure has been reduced even to a few millimetres.

Thus Macewen had performed the operation of removal of the whole lung as long ago as 1897. Yet his pioneer work was almost forgotten until the War came, when surgeons again discovered that the physics of lung expansion were all wrong and intrathoracic operations were performed as freely as intra-abdominal. No one perhaps played a more important part in putting the bogey of differential pressure to flight than Professor Pierre Duval of Paris, whose "War Wounds of the Lung"[1] was the one surgical classic which the Great War brought forth. It was Duval who, in 1916, said: "The lung is no longer the dreaded organ it was before the War. . . . Surgery of the lung takes its place amongst the recognized procedures of general surgery." But even Duval seems to have forgotten Macewen's dramatic exposition at the Congress in 1913, and his Cavendish Lecture in 1906.[2] Professor Sauerbruch in his Macewen Lecture[3] delivered at Glasgow, paid a generous tribute to Macewen's pre-eminence in thoracic surgery.

[1] Duval. *War Wounds of the Lung.* John Wright & Sons, Bristol, 1918.

[2] Macewen. "The Cavendish Lecture on Some Points on the Surgery of the Lung." *West London Medical Journal,* July, 1906.

[3] Sauerbruch. "Macewen Memorial Lecture," 1937. Glasgow University Press, 1937.

**"A Woman's Calling."**

THE National Association of Local Government Officers has published a brochure, "A Woman's Calling," which certainly deserves the notice of those interested in Nursing and Public Health Services.

Since the Lancet Commission on Nursing issued its report in 1932 the Association has been devising a scheme for recruitment and training of women engaged in the Public Health Services. This brochure explains the scheme and as it deals very fully with the Nursing Service no apology is needed for referring to some of the proposals in this *Journal*.

If the suggestions regarding nurses' hours of duty, freedom from oppressive regulations, and pay, were carried out, one imagines there would be little difficulty in getting suitable girls and women to undertake the Nursing and Public Health Services.

It appears that the suggestion of hours on duty which total 96 hours a fortnight can be arranged more easily than the suggested 48-hour week, and would not be unduly long. One day a week off duty and four weeks' holiday a year would be generous treatment: one wonders if this is possible.

As regards freedom from oppressive regulations, after the first year's probationership, discipline and orderly behaviour should have been sufficiently inculcated to allow such regulations to be foregone and to permit freedom for recreation in off-duty time without annoying restrictions. If a girl or a woman takes an appointment as clerk in a Public Health Department her free time is her own; there seems no reason why a girl who has to do with matters of life and death should be considered unworthy of the same treatment. The regulations which press so heavily on the modern nurse are a legacy from the days when convents and monasteries were the only institutions in which the sick were tended. Moreover, Florence Nightingale's rules were undoubtedly very harsh.

As regards pay the scale proposed is really generous, but it might be contended that such pay would delate the voluntary hospitals' nursing service completely; that is, if money were the chief thing concerned.

Among the objectives set forth are certain reforms which the Association desires to urge upon the General Nursing Councils. These reforms are of a kind to enlist a considerable amount of support from the medical profession:—

(*a*) Divide the preliminary state examination into two parts: Part I, Anatomy, Physiology and Hygiene; and Part II, Theory and Practice of Nursing.

(*b*) Agree to the examination for Part I being taken prior to entry for general training.

(*c*) Require all hospitals recognized as complete training schools to admit probationer nurses at age 17–18 years to a three years' course.

(*d*) Require all hospitals recognized as complete training schools to make an allowance for time spent in any form of approved preliminary training, provided satisfactory evidence of such training is produced.

(*e*) Limit the questions in the final state examinations for all parts of the register to nursing treatment; neither systematic medicine, surgery, gynæcology or psychiatry to be included.

**Death of Mr. Hartland Wright.**

THE death of Mr. Hartland S. Wright, in his 90th year, removes one of the outstanding figures in the Medical Publishing world, of whom the city of Bristol may be justly proud. For many years he was the Chairman of Messrs. John Wright & Sons, Ltd., which was one of the first provincial publishing houses to undertake medical work.

The two outstanding enterprises which have been undertaken in the present generation by this firm have been the *Medical Annual* and the *British Journal of Surgery.* Both these publications have been of very great importance to the medical world. The first has presented an up-to-date résumé of medical progress year by year, which has been invaluable to busy practitioners, anxious to keep abreast of the times. The second has established an authoritative journal, representative of British surgery, including that of the Dominions and Colonies. Although for some years past Mr. Hartland Wright's health precluded his active participation in business, his influence and personality will long remain as the inspiring model of enterprise and efficiency which have become the watchwords of the firm.

